# Demographic and Social Factors Associated with COVID-19 Vaccination Initiation Among Adults Aged ≥65 Years — United States, December 14, 2020–April 10, 2021

**DOI:** 10.15585/mmwr.mm7019e4

**Published:** 2021-05-14

**Authors:** Ari Whiteman, Alice Wang, Kelly McCain, Betsy Gunnels, Robin Toblin, James Tseryuan Lee, Carolyn Bridges, Laura Reynolds, Bhavini Patel Murthy, Judy Qualters, James A. Singleton, Kimberley Fox, Shannon Stokley, LaTreace Harris, Lynn Gibbs-Scharf, Neetu Abad, Kathryn A. Brookmeyer, Susan Farrall, Cassandra Pingali, Anita Patel, Ruth Link-Gelles, Sharoda Dasgupta, Radhika Gharpure, Matthew D. Ritchey, Kamil. E. Barbour

**Affiliations:** ^1^CDC COVID-19 Response Team; ^2^Geospatial Research, Analysis, and Services Program, Agency for Toxic Substances and Disease Registry, Atlanta, Georgia.

Compared with other age groups, older adults (defined here as persons aged ≥65 years) are at higher risk for COVID-19–associated morbidity and mortality and have therefore been prioritized for COVID-19 vaccination (*1*,*2*). Ensuring access to vaccines for older adults has been a focus of federal, state, and local response efforts, and CDC has been monitoring vaccination coverage to identify and address disparities among subpopulations of older adults (*2*). Vaccine administration data submitted to CDC were analyzed to determine the prevalence of COVID-19 vaccination initiation among adults aged ≥65 years by demographic characteristics and overall. Characteristics of counties with low vaccination initiation rates were quantified using indicators of social vulnerability data from the 2019 American Community Survey.* During December 14, 2020–April 10, 2021, nationwide, a total of 42,736,710 (79.1%) older adults had initiated vaccination. The initiation rate was higher among men than among women and varied by state. On average, counties with low vaccination initiation rates (<50% of older adults having received at least 1 vaccine dose), compared with those with high rates (≥75%), had higher percentages of older adults without a computer, living in poverty, without Internet access, and living alone. CDC, state, and local jurisdictions in partnerships with communities should continue to identify and implement strategies to improve access to COVID-19 vaccination for older adults, such as assistance with scheduling vaccination appointments and transportation to vaccination sites, or vaccination at home if needed for persons who are homebound.^†^ Monitoring demographic and social factors affecting COVID-19 vaccine access for older adults and prioritizing efforts to ensure equitable access to COVID-19 vaccine are needed to ensure high coverage among this group.

COVID-19 vaccine administration data are reported to CDC by multiple entities using immunization information systems, the Vaccine Administration Management System, pharmacy systems, or direct submission of electronic health records.^§^ Vaccination initiation rates were estimated as the percentage of older adult residents who received at least 1 dose of COVID-19 vaccine during December 14, 2020–April 10, 2021, and whose data were reported to CDC by April 16, 2021.^¶^ Vaccination initiation rates by age group (65–74 or ≥75 years) and sex, nationally and by state,** were estimated by dividing demographic data reported for each vaccine recipient by population estimates for adults aged ≥65 years from the U.S. Census Bureau 2019 Population Estimates Program.^††^ Analyses of vaccination initiation rates by race/ethnicity of the vaccine recipient were conducted at the national level only (because of a high level of missing data by state and low sample size for some race/ethnicity groups) and are presented as the percentage of total adults aged ≥65 years with known race/ethnicity information in each race/ethnicity category.

For county-level analyses, five frequent indicators of social vulnerability (*3*,*4*) for older adults were gathered from the U.S. Census Bureau American Community Survey 5-year estimates from 2019: the percentage of older adult county residents 1) without a computer (e.g., desktop or laptop computer [excludes mobile phones]); 2) with a computer but without Internet access; 3) living alone; 4) having an annual income below the federal poverty level; and 5) identifying as a person with race/ethnicity other than non-Hispanic White (White) alone. A generalized estimating equation for each of these social vulnerability indicators was used with a normal distribution and identity link function to quantify and compare the average percentage of older adults with social vulnerabilities in each county to the county vaccination initiation rate, divided into initiation categories of <50%, 50% to <75%, and ≥75%.^§§^ SAS software (version 9.4; SAS Institute) was used to conduct analyses. This activity was reviewed by CDC and was conducted consistent with applicable federal law and CDC policy.^¶¶^

During December 14, 2020–April 10, 2021, a total of 42,736,710 older adult residents of 50 states and the District of Columbia received at least 1 dose of COVID-19 vaccine (Table). The rate of vaccination initiation was 79.1% overall and, by jurisdiction, ranged from 68.9% (Alabama) to 99.9% (New Hampshire). Nationally, the rate of vaccination initiation was 1.3 percentage points higher among persons aged 65–74 years (79.6%) than among persons aged ≥75 years (78.3%). Among persons aged 65–74 years, vaccination initiation rates ranged from 66.8% (Alabama) to 99.9% (New Hampshire), and among persons aged ≥75 years, ranged from 69.1% (Mississippi) to 99.9% (New Hampshire) (Figure 1). Nationally, the vaccination initiation rate was 2.1 percentage points higher among men (79.6%) than among women (77.5%).***

**TABLE:** COVID-19 vaccination initiation rate among adults aged ≥65 years, by state, age group, and sex* — United States, December 14, 2020–April 10, 2021

Jurisdiction	No. of persons with at least 1 COVID-19 vaccine dose (% vaccination initiation)				
	Total men and women aged ≥65 years	Age group, yrs		Sex	
		65–74	≥75	Men	Women
**National total**	42,736,710 (79.1)	**25,051,017 (79.6)**	**17,685,693 (78.3)**	**19,166,329 (79.6)**	**23,252,363 (77.5)**
Alabama	585,732 (68.9)	334,760 (66.8)	250,972 (72.0)	260,221 (70.1)	324,554 (67.8)
Alaska	70,003 (76.4)	47,306 (77.2)	22,697 (74.9)	34,965 (76.9)	34,306 (74.4)
Arizona	991,737 (75.8)	568,864 (75.7)	422,873 (75.9)	460,031 (76.4)	528,908 (74.9)
Arkansas	372,077 (71.0)	210,948 (69.7)	161,129 (72.9)	165,732 (71.0)	200,562 (69.1)
California	4,939,416 (84.6)	2,934,905 (86.7)	2,004,511 (81.8)	2,228,999 (85.7)	2,690,716 (83.2)
Colorado	677,637 (80.4)	415,844 (79.6)	261,793 (81.7)	312,828 (80.9)	363,544 (79.8)
Connecticut	549,241 (87.1)	308,545 (87.5)	240,696 (86.8)	242,538 (87.9)	305,959 (86.4)
Delaware	159,934 (84.7)	96,468 (85.5)	63,466 (83.5)	71,975 (85.1)	87,258 (83.6)
District of Columbia	65,115 (74.6)	39,254 (78.2)	25,861 (69.7)	27,615 (77.3)	37,141 (72.0)
Florida	3,632,615 (80.8)	2,037,253 (82.6)	1,595,362 (78.5)	1,657,775 (81.7)	1,957,688 (79.3)
Georgia	1,114,150 (73.4)	679,273 (73.4)	434,877 (73.6)	483,369 (73.1)	608,901 (71.2)
Hawaii	208,887 (77.8)	115,405 (75.9)	93,482 (80.3)	96,534 (79.4)	110,985 (75.6)
Idaho	216,332 (74.4)	127,441 (72.5)	88,891 (77.4)	101,554 (73.7)	112,489 (73.6)
Illinois	1,641,523 (80.3)	955,599 (81.3)	685,924 (79.1)	726,643 (81.2)	908,243 (79.1)
Indiana	822,469 (75.8)	481,217 (75.6)	341,252 (76.0)	370,554 (77.1)	450,399 (74.4)
Iowa	458,859 (83.0)	260,017 (83.5)	198,842 (82.3)	204,719 (82.3)	248,043 (81.5)
Kansas	408,580 (85.9)	234,751 (86.2)	173,829 (85.5)	183,897 (86.0)	223,731 (85.5)
Kentucky	583,439 (77.7)	349,547 (78.0)	233,892 (77.3)	262,481 (78.8)	318,951 (76.4)
Louisiana	539,780 (72.8)	323,992 (73.2)	215,788 (72.3)	241,328 (74.1)	297,074 (71.5)
Maine	252,223 (88.4)	150,338 (88.9)	101,885 (87.7)	116,099 (89.3)	135,291 (87.1)
Maryland	771,161 (80.4)	452,878 (80.6)	318,283 (80.0)	337,410 (81.3)	429,662 (78.9)
Massachusetts	1,025,207 (87.7)	588,874 (87.5)	436,333 (88.0)	445,646 (87.6)	570,880 (86.4)
Michigan	1,333,607 (75.5)	787,506 (75.9)	546,101 (75.0)	606,985 (76.7)	725,509 (74.5)
Minnesota	784,098 (85.2)	452,519 (85.2)	331,579 (85.2)	357,349 (85.2)	419,766 (83.8)
Mississippi	337,396 (69.3)	200,815 (69.5)	136,581 (69.1)	149,898 (70.7)	186,511 (67.9)
Missouri	777,239 (73.2)	446,746 (73.2)	330,493 (73.1)	349,730 (74.4)	426,141 (72.0)
Montana	156,168 (75.6)	92,439 (74.2)	63,729 (77.9)	74,335 (75.1)	80,694 (75.1)
Nebraska	255,621 (81.8)	146,664 (82.0)	108,957 (81.6)	115,091 (81.7)	137,046 (79.9)
Nevada	366,508 (73.9)	221,813 (73.1)	144,695 (75.1)	173,614 (74.3)	192,185 (73.2)
New Hampshire	275,371 (99.9)	168,361 (99.9)	107,010 (99.9)	124,620 (99.9)	144,939 (99.9)
New Jersey	1,178,070 (79.8)	678,214 (81.4)	499,856 (77.8)	513,465 (80.5)	659,877 (78.8)
New Mexico	311,667 (82.5)	183,263 (81.6)	128,404 (83.9)	142,292 (82.6)	168,281 (81.9)
New York	2,424,208 (73.5)	1,416,044 (76.1)	1,008,164 (70.2)	1,063,736 (74.8)	1,329,168 (71.0)
North Carolina	1,308,317 (74.7)	785,219 (75.0)	523,098 (74.2)	580,067 (75.7)	717,019 (72.8)
North Dakota	95,434 (79.6)	53,736 (80.6)	41,698 (78.4)	43,160 (78.0)	49,043 (76.0)
Ohio	1,561,494 (76.3)	907,395 (76.3)	654,099 (76.3)	686,703 (76.1)	849,450 (74.2)
Oklahoma	494,734 (77.9)	288,388 (78.4)	206,346 (77.3)	223,789 (78.8)	270,043 (76.9)
Oregon	588,122 (76.8)	349,384 (75.2)	238,738 (79.2)	269,639 (76.8)	316,839 (76.4)
Pennsylvania	2,084,215 (87.1)	1,208,999 (89.3)	875,216 (84.2)	900,086 (85.6)	1,131,953 (84.4)
Rhode Island	164,945 (88.2)	95,963 (90.4)	68,982 (85.3)	72,707 (89.3)	91,967 (87.0)
South Carolina	726,525 (77.5)	432,172 (75.7)	294,353 (80.3)	327,860 (78.7)	397,494 (76.4)
South Dakota	131,960 (86.9)	74,675 (83.7)	57,285 (91.4)	59,261 (83.9)	69,307 (85.3)
Tennessee	806,104 (70.5)	465,412 (68.3)	340,692 (73.7)	362,397 (71.6)	441,628 (69.3)
Texas	2,801,138 (75.0)	1,694,786 (75.5)	1,106,352 (74.3)	1,250,589 (74.9)	1,519,330 (73.6)
Utah	297,419 (81.3)	176,259 (80.3)	121,160 (82.8)	137,974 (80.9)	155,667 (79.7)
Vermont	116,107 (92.9)	69,807 (92.8)	46,300 (92.9)	54,190 (94.1)	61,845 (91.7)
Virginia	1,089,519 (80.2)	642,006 (80.0)	447,513 (80.4)	489,594 (81.2)	597,041 (79.0)
Washington	1,002,812 (82.9)	604,837 (82.2)	397,975 (83.9)	458,165 (83.0)	537,814 (81.8)
West Virginia	253,825 (69.2)	148,838 (68.5)	104,987 (70.1)	117,940 (70.5)	134,669 (67.5)
Wisconsin	857,203 (84.3)	502,468 (84.5)	354,735 (84.0)	393,780 (84.6)	459,800 (83.3)
Wyoming	70,767 (71.4)	42,810 (70.7)	27,957 (72.4)	34,400 (72.0)	36,052 (70.1)

* Information on sex was missing for 318,018 (0.74%) vaccine recipients.

**FIGURE 1:**
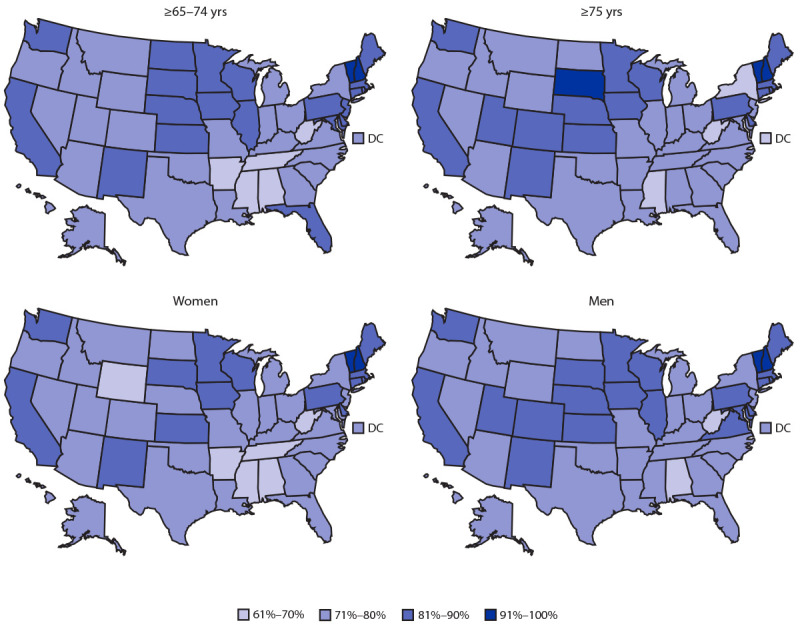
State COVID-19 vaccination initiation rate of adults aged ≥65 years, by age group and sex — United States, December 14, 2020–April 10, 2021 **Abbreviation:** DC = District of Columbia.

Among recipients of at least 1 dose of COVID-19 vaccine, race/ethnicity was missing in 17,903,625 (41.9%) records. Among the 24,833,085 recipients with reported race/ethnicity, 17,561,065 (70.7%) were White; 1,885,433 (7.6%) were non-Hispanic Black; 1,548,776 (6.2%) were non-Hispanic multiracial; 1,657,517 (6.7%) were Hispanic; 880,040 (3.5%) were non-Hispanic Asian; 190,856 (0.8%) were non-Hispanic American Indian or Alaskan Native; 42,747 (0.2%) were Native Hawaiian or Other Pacific Islander; and 1,066,651 (4.3%) identified as “all other races/ethnicities.”

Counties with <50% vaccination initiation rates had significantly higher average percentages of older adults with social vulnerabilities than did counties with vaccination initiation rates ≥75%, with the exception of the race/ethnicity other than White indicator (Figure 2). In counties with <50% vaccination initiation rates, an average of 24.6% (95% confidence interval [CI] = 22.3%–26.9%) of older adults did not have a computer, compared with 19.1% (95% CI = 17.8%–20.4%) in counties with ≥75% vaccination initiation rates. Similarly, the average percentage of older adults without Internet access was 9.9% (95% CI = 8.9%–10.9%) in counties with <50% vaccination initiation rates, compared with 7.4% (95% CI = 7.0%–7.8%) in counties with ≥75% vaccination initiation rates. The average percentage of older adults living in poverty was 10.3% (95% CI = 9.2%–11.4%) in counties with <50% vaccination initiation rates, compared with 7.6% (95% CI = 7.0%–8.2%) in counties with ≥75% vaccination initiation rates. The average percentage of older adults living alone was 14.3% (95% CI = 13.8%–14.9%) in counties with <50% vaccination initiation rates, compared with 12.2% (95% CI = 11.8%–12.6%) in counties with ≥75% vaccination initiation rates. The average percentage of older adults indicating race/ethnicity other than White was similar in counties with <50% vaccination initiation rates (8.0%; 95% CI = 4.9%–11.1%) and ≥75% vaccination initiation rates (9.3%; 95% CI = 6.4%–12.1%).

**FIGURE 2:**
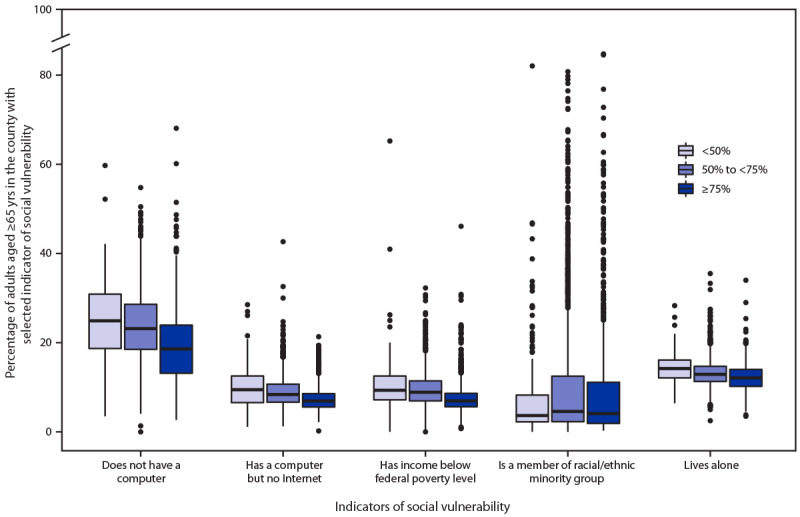
County residents aged ≥65 years with selected indicators of social vulnerability, by vaccination initiation percentage — United States, December 14, 2020–April 10, 2021* **Abbreviation:** IQR = interquartile range. * This figure presents boxplots with the distributions of each indicator of social vulnerability for each of the categories of vaccination initiation among the population aged ≥65 years. The horizontal line in each box indicates the median; the top and bottom edges of each box indicate the 75th and 25th percentile values, respectively; the top and bottom of each vertical line show the maximum (75th percentile value + 1.5 x IQR) and minimum (25th percentile value - 1.5 x IQR); the dots represent outliers for each distribution.

## Discussion

Among adults aged ≥65 years in the United States, 79.1% had initiated COVID-19 vaccination as of April 10, 2021. Despite COVID-19 vaccine becoming available on December 14, 2020, and many states including older adults among the first groups eligible for vaccination, as many as 11.3 million older adults remained unvaccinated as of April 10, 2021.^†††^ Further, vaccination initiation was lower among certain demographic groups (e.g., women) and states, and on average, counties with lower vaccination initiation rates had higher percentages of older adults with social vulnerabilities.

As of April 10, 2021, vaccination initiation rates among older adults nationwide were higher among men and persons aged 65–74 years than among women and persons aged ≥75 years. In comparison, according to reports of recent estimates, no differences by sex have been observed in initiation of vaccination with influenza and shingles vaccine, both of which are also recommended for older adults.^§§§^ Vaccination acceptance by age group and sex will be followed to determine whether these differences persist as vaccine availability expands.

Initiation overall and by demographic subpopulation varied by state, indicating that national-level analyses might obscure more local trends. New Hampshire and Vermont had the two highest overall vaccination initiation rates among older adults. New Hampshire established early partnerships with pharmacies, first responders, and the National Guard, in addition to creating a centralized state website for vaccination sign-up. In Vermont, state health authorities established a partnership to coordinate vaccine distribution and administration with the Association of Hospitals and Health Systems, which represents Vermont's 14 nonprofit hospitals. At the county level, the upper Midwest (e.g., Iowa, Minnesota, and Wisconsin) reported high vaccination initiation rates as well, relative to other regions.

In addition to differences in vaccination initiation rates by age group and sex identified at the state level, counties with <50% initiation rates, on average, included higher percentages of older adults experiencing social vulnerabilities than did counties with ≥75% initiation. The identification of higher prevalence of older adults with social vulnerabilities in counties with low relative vaccination initiation rates is consistent with previous disparities identified among older adults who had received the shingles vaccine and among all adults who had received the COVID-19 vaccine.^¶¶¶^ Given the increased risk for COVID-19–related morbidity and mortality among older adults, addressing COVID-19 vaccine access barriers for socially vulnerable communities of older adults is critical. Among older adults, issues such as loneliness or absence of regular companionship (*5*), lack of computer or Internet literacy (*6*), and limited transportation options might be addressed through specialized outreach and vaccine distribution programs, as jurisdictions such as Miami, Florida, and Fulton County, Georgia, have demonstrated (*7*,*8*). In some states, such as Texas and Pennsylvania, state health departments and vaccination providers have formed partnerships with interest groups and community-based organizations to create programs designed to guide older adults through the vaccination sign-up process and transport them to vaccination sites (*9*,*10*).

The findings in this report are subject to at least five limitations. First, persons who were vaccinated through the Pharmacy Partnership for Long-Term Care Program**** were not analyzed separately. These persons, whose vaccinations were arranged and administered by pharmacy partners at their residential facilities, might not face access barriers similar to those experienced by persons in other residential settings. However, an estimated 95% of Medicare beneficiaries (who constitute an estimated 96% of older adults) reside in the community rather than long-term care facilities.^††††^ Second, associations between county social vulnerability and vaccination initiation rates are ecological and reported for population-based indicators rather than individual-level vulnerability. Third, vulnerabilities and vaccination initiation rates might vary within counties because state and local jurisdictions might prioritize vaccination efforts for communities of older adults in smaller geographic units (e.g., ZIP codes). Fourth, older adult health and health care access are associated with numerous additional indicators for which recent data at the county level are not available. These additional indicators, such as living in multigenerational households or limitations accessing public transportation, might be associated with unexplained variance in the models. Finally, given that vaccine administration data are reported to CDC by multiple entities using various data systems, the possibility of underreporting, and thus, underestimation of vaccination coverage cannot be ruled out.

As COVID-19 vaccine supply expands along with the individual eligibility criteria, state and local jurisdictions can continue to ensure that older adults have equitable access to COVID-19 vaccines, including assistance with scheduling vaccination appointments and transportation to vaccination sites, or vaccination at home if needed for persons who are homebound. Assistance to ensure that persons receiving a vaccine that requires 2 doses to complete the series might be needed as well. Public health officials should continue to monitor vaccination initiation rates in the context of socioeconomic and demographic vulnerability to promote vaccine administration among this population at high risk for severe illness and death from COVID-19.

**Summary**

**What is already known about this topic?**

Older adults have experienced higher risk for COVID-19–associated morbidity and mortality and therefore have been prioritized for COVID-19 vaccination.

**What is added by this report?**

After the first 3.5 months of the U.S. COVID-19 vaccination program, 79.1% of adults aged ≥65 years had received ≥1 dose, with higher vaccination initiation among men. Counties with lower vaccination initiation rates had higher percentages of older adults with social vulnerabilities.

**What are the implications for public health practice?**

Monitoring demographic and social factors affecting COVID-19 vaccine access for older adults and prioritizing efforts to ensure equitable access to COVID-19 vaccine are needed to ensure high coverage among this group.

## Footnotes

*
https://www.census.gov/programs-surveys/acs

† https://www.cdc.gov/vaccines/covid-19/clinical-considerations/homebound-persons.html; https://www.cdc.gov/vaccines/covid-19/clinical-considerations/older-adults-and-disability/access.html

§ https://www.cdc.gov/vaccines/covid-19/vaccination-provider-support.html; https://www.cdc.gov/vaccines/covid-19/reporting/vams/index.html

¶ Providers are required to report administration records to the state immunization information system within 72 hours; 5 additional days of observation were included to account for delays in reporting and transmission of records to CDC. Data include all COVID-19 vaccine manufacturers, including the Janssen (Johnson & Johnson) vaccine, which only requires a single dose.

** Exclusions from the state-level dataset include 1) recipients for whom state of residence was unknown (48,524); 2) recipients aged ≥65 years for whom specific age (36,153) or sex (318,018) was unknown; and 3) residents of eight U.S. territories and freely associated states and armed forces overseas (409,206) for which population denominator data were not available.

†† Exclusions from the county-level dataset include 1) recipients for whom county of residence was unknown (2,406,423) or was known yet reported with an invalid state of residence (70,955); 2) recipients for whom state of residence was unknown (28,313); 3) residents of eight U.S. territories, freely associated states, and armed forces overseas (405,139) for which population denominator data were not available, and after excluding based on the above criteria; 4) residents of Delaware and the District of Columbia because each has three or fewer counties (218,240); and 5) residents of Georgia, Hawaii, South Dakota, and West Virginia because of unknown county of residence in >20% of records (776,207).

§§
https://www.census.gov/data/datasets/time-series/demo/popest/2010s-counties-total.html

¶¶ 45 C.F.R. part 46, 21 C.F.R. part 56; 42 U.S.C. Sect. 241(d); 5 U.S.C. Sect. 552a; 44 U.S.C. Sect. 3501 et seq.

*** Among recipients aged ≥65 years, sex was missing in 318,018 of records.

††† The U.S. Census Bureau estimates the population of older adults to be 54,058,263. With 42,736,710 older adults receiving ≥1 COVID-19 vaccine dose, an estimated 11,321,553 older adults remained unvaccinated as of April 10, 2021.

§§§ https://www.cdc.gov/nchs/products/databriefs/db370.htm; https://www.cdc.gov/flu/fluvaxview/coverage-1920estimates.htm

¶¶¶ https://www.cdc.gov/mmwr/volumes/70/wr/mm7012e1.htm?s_cid=mm7012e1_w; https://www.cdc.gov/nchs/products/databriefs/db370.htm

****
https://www.cdc.gov/vaccines/covid-19/long-term-care/pharmacy-partnerships.html

††††
https://www.agingstats.gov/docs/LatestReport/OA20_EmbargoCopy.pdf
